# Caloric restriction reduces the systemic progression of mouse AApoAII amyloidosis

**DOI:** 10.1371/journal.pone.0172402

**Published:** 2017-02-22

**Authors:** Lin Li, Jinko Sawashita, Xin Ding, Mu Yang, Zhe Xu, Hiroki Miyahara, Masayuki Mori, Keiichi Higuchi

**Affiliations:** 1 Department of Aging Biology, Institute of Pathogenesis and Disease Prevention, Shinshu University Graduate School of Medicine, Matsumoto, Japan; 2 Department of Biological Sciences for Intractable Neurological Diseases, Institute for Biomedical Sciences, Interdisciplinary Cluster for Cutting Edge Research, Shinshu University, Matsumoto, Japan; 3 Department of Advanced Medicine for Health Promotion, Institute for Biomedical Sciences, Interdisciplinary Cluster for Cutting Edge Research, Shinshu University, Matsumoto, Japan; Universita degli Studi di Padova, ITALY

## Abstract

In mouse senile amyloidosis, apolipoprotein (Apo) A-II is deposited extracellularly in many organs in the form of amyloid fibrils (AApoAII). Reduction of caloric intake, known as caloric restriction (CR), slows the progress of senescence and age-related disorders in mice. In this study, we intravenously injected 1 μg of isolated AApoAII fibrils into R1.P1-*Apoa2c* mice to induce experimental amyloidosis and investigated the effects of CR for the next 16 weeks. In the CR group, AApoAII amyloid deposits in the liver, tongue, small intestine and skin were significantly reduced compared to those of the *ad libitum* feeding group. CR treatment led to obvious reduction in body weight, improvement in glucose metabolism and reduction in the plasma concentration of ApoA-II. Our molecular biological analyses of the liver suggested that CR treatment might improve the symptoms of inflammation, the unfolded protein response induced by amyloid deposits and oxidative stress. Furthermore, we suggest that CR treatment might improve mitochondrial functions via the sirtuin 1-peroxisome proliferator-activated receptor γ coactivator 1α (SIRT1-PGC-1α) pathway. We suggest that CR is a promising approach for treating the onset and/or progression of amyloidosis, especially for systemic amyloidosis such as senile AApoAII amyloidosis. Our analysis of CR treatment for amyloidosis should provide useful information for determining the cause of amyloidosis and developing effective preventive treatments.

## Introduction

Amyloidosis is a group of diseases characterized by extracellular or intracellular deposition of insoluble amyloid fibrils [1,2]. Fibrils are formed when normally soluble proteins aggregate due to conformational changes caused by various mechanisms. Amyloidoses share several common properties. That is, amyloid fibrils have a characteristic ultrastructural appearance and a β-pleated sheet core structure regardless of the amyloidosis-associated protein. Amyloid fibrils and oligomers of aggregates cause profound dysfunction in both cells and tissues, and these lead to a number of diseases [3,4]. Currently, over 30 amyloid fibril proteins are known to be associated with amyloidosis [2,5]. In some types of amyloidosis, such as Alzheimer’s disease, prion diseases, and type II diabetes, amyloid deposits are limited to a unique organ such as the brain or pancreas. In systemic amyloidoses, including amyloid light chain amyloidosis, familial amyloid polyneuropathy, reactive amyloid A (AA) amyloidosis and dialysis-related amyloidosis, amyloid fibrils deposit in multiple organs such as the liver, kidney, heart, lung and spleen.

Apolipoprotein (Apo) A-II is the second most abundant apolipoprotein in serum high-density lipoprotein (HDL) in humans and mice, but its roles in HDL are still unclear [6]. Studies suggest that ApoA-II might be an important modulator of reverse cholesterol transport and an HDL protein that helps protect low-density lipoprotein against oxidation [6–8]. It may also be associated with susceptibility to atherosclerosis [6]. We found that ApoA-II accumulates to form amyloid fibrils (AApoAII) that deposit extracellularly in various organs with aging [9, 10]. AApoAII amyloidosis is found in both humans and mice, but there are some differences. In humans, it is due to a mutation in the normal stop codon in the ApoA-II gene and it has been observed mainly in the kidneys [11,12]. In aged mice of many strains, it has been observed systemically in several organs (excluding the brain) [13,14]. ApoA-II amino acid sequences of humans and mice differ by approximately 40% and they exist in different forms: a disulfide-homodimer in humans and a monomer in mice. However, both ApoA-II proteins exist mainly in HDL particles and they may have similar roles [6].

Among strains of laboratory mice, six alleles (*a*, *b*, *c*, *d*, *e* and *f*) of the *Apoa2* gene have been found [15]. Several analyses have shown that the *Apoa2c* allele codes for the most amyloidogenic ApoA-II [10,16,17]. We have reported that intravenous, intraperitoneal and oral administration of a very small amount of AApoAII fibrils markedly accelerated amyloid deposits in young mice [18–20]. Intriguingly, our recent studies have suggested that AApoAII amyloidosis was transmissible by a prion-like infectious process through a seeding-nucleation mechanism [17,20–24]. These findings have suggested that mouse AApoAII amyloidosis is an extremely useful model for the analysis of systemic amyloidoses and the development of new preventive treatments for amyloidoses.

In amyloidoses, especially age-associated amyloidosis including Alzheimer’s disease, type II diabetes, familial amyloid polyneuropathy and AA amyloidosis, preventive treatments before/after early diagnosis have become a pressing issue. Nutritional control and caloric restriction (CR) may be the most readily available treatment to prevent or slow these amyloidoses [25–28]. In particular, CR, i.e., a ~60% reduction of intake compared to an *ad libitum* (AL) diet, has been reported to be the most effective non-genetic treatment to decelerate aging and extend life- and health-span [26–29].

The molecular mechanisms by which longevity is promoted by CR intervention are complex. One important metabolic reaction mediated by CR is autophagy [30,31]. This process supplies organisms with nutrients via the cytoplasmic recycling system [30]. It also maintains damaged organelles and proteins during aging and increases longevity. Overexpression of ATG5, which is a key molecule of autophagy, extended lifespan in mice [31]. However, there are few proteomic studies of CR in mammals during their growth phase. A recent report revealed that high quality protein was initially maintained in the livers of young C57BL/6 mice after short-term CR through a reduction of synthetic burden and metabolic damage to protein. A treatment with rapamycin, a reagent that mimics CR, might have different mechanisms [32]. For example, CR extended the mean half-lives of short-lived proteins of canonical pathways such as LXR/RXR activation, including ApoA-I and ApoA-II, acute phase response, fatty acid β-oxidation and mitochondrial proteins. In contrast, rapamycin did not show these effects. Another recent report revealed that several chaperones in the livers of CR-treated mice were either unchanged or reduced in a manner similar to rapamycin-treated mice [33]. Importantly, chaperons are required in general for remodeling of misfolded proteins that increase with aging and/or age-related diseases. In contrast, a very recent report demonstrated that redox-dependent recruitment of chaperones by CR maintains proteostasis and that disaggregates of misfolded proteins increased with aging and led to an extended lifespan in yeast [34].

It has been suggested that CR could be a good intervention for improving age-related diseases such as type-II diabetes, hypertension, atherosclerosis and dementia [25,35–37]. For example, CR improves type II diabetes by altering the lipid droplet proteome and diacylglycerol species in the liver of NZO mice, a model of type II diabetes [37]. In contrast, a high fat diet increases the risk of Alzheimer’s disease in humans and exacerbates pathological depositions of AA protein, islet amyloid precursor protein and β-amyloid in an amyloidosis model in mice [38–41]. It is known that CR mitigates neuropathology compared with AL feeding in several mouse models of Alzheimer’s disease [42–44].

The underlying mechanisms by which CR treatment mitigates Alzheimer’s disease are suggested by a number of observations. First, both circulating insulin and insulin signaling are altered by CR treatment, enhancing the degradation of β-amyloid via enzymatic processes [25,42]. Second, CR treatment activates sirtuin-1 (SIRT1) signaling and enhances the function of non-amyloidogenic processing enzyme of the amyloid precursor protein [43]. Third, autophagy induced by CR treatment appears to suppress the progression of Alzheimer’s disease [45]. In this regard, there are two reports that demonstrated that activated autophagy degraded amyloid fibrils or reduced levels of amyloid β peptide and amyloid precursor protein [46,47]. In AApoAII amyloidosis, we previously reported that chronic CR (60% caloric intake compared with an AL group) decelerated the advancement of senescence in SAMP1 mice and inhibited the spontaneous deposition of AApoAII fibrils with aging. However, the mechanisms reducing amyloidosis were unclear [48]. Here, we hypothesize that CR treatment does indeed play a preventive role against the progression of systemic amyloidosis. Moreover, we demonstrate that CR treatment reduced the progression of amyloidosis in mice with inducible systemic AApoAII amyloidosis. A possible molecular mechanism is discussed.

## Materials and methods

### Animals

We used R1.P1-*Apoa2c* congenic mice in all experiments. This strain is a useful model for analysis of systemic AApoAII amyloidosis [49]. It carries the amyloidogenic *Apoa2c* allele from the AApoAII amyloidosis-susceptible SAMP1 strain on a genetic background of the Senescence-Accelerated mouse Resistant 1 (SAMR1) strain, which has a normal aging process. Moreover, AApoAII amyloidosis can be induced early and systemically and with certainty regardless of gender after a single injection of AApoAII fibrils [14,18–20]. They were developed in our laboratory and have been maintained in the Division of Animal Research, Research Center for Supports to Advanced Science, Shinshu University. We have used sister-brother mating under specific pathogen-free conditions at 24 ± 2°C with a light-controlled regimen (12 h light/dark cycle, lights on at 9:00 and off at 21:00). Mice were maintained on a commercial diet (MF, Oriental Yeast, Tokyo, Japan) and tap water and were rehoused in cleaned cages (W200 x L300 x H100 mm) every Thursday morning (approximately from 9:30 to 10:30).

Multiple female mice were housed in a single cage for analyses of AApoAII amyloidosis. The reasons for this strategy are as follows. First, mice generally show group-living behavior throughout their lives, and housing of individual animals would cause inappropriate stress. Second, mouse AApoAII amyloidosis occurs in several organs independent of gender [14]. Third, it was important to avoid the possibility that the experimental results might be confounded by AA amyloid deposition and/or other adverse impacts due to fighting among male mice housed in the same cage. Female mice born in August (first series) or September (second series) of 2013 were selected and housed 6 to 8 per cage at 3 weeks of age and were given commercial diet and tap water freely until the beginning of the experiments.

**Fig 1:**
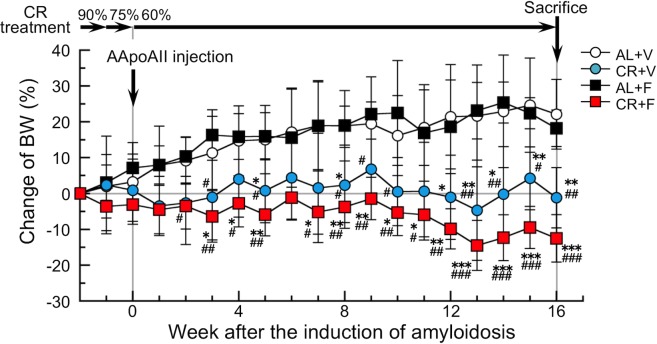
Experimental design and percent changes of BWs in mice from baseline during the CR intervention. CR treatment was performed by a step-down protocol to reach 60% of AL feeding levels, after which it was continued for 16 weeks. The induction of AApoAII amyloidosis in the AL+F and CR+F groups was performed when 60% CR was started. Each symbol and bar represents the mean ± S.D. (N = 6 (CR+F) or 5 (others)). P values were obtained by the Tukey-Kramer method for multiple comparison at corresponding time. *p<0.05, **p<0.01, ***p<0.001 vs. the AL+V group. #p<0.05, ##p<0.01, ###p<0.001 vs. the AL+F group.

At 8 weeks of age, each of 16 mice in the two series was randomly assigned into AL feeding or CR groups to avoid significant difference in the body weights (BWs) between the groups and were housed 4 per cage without regrouping (S1 Table and S1 Fig). Food intake of the AL group was determined weekly between Monday and Wednesday. The intake of the CR group was adjusted weekly according to the intake of the AL group over the preceding week. The CR groups were fed daily (Monday-Friday) and 2-day amounts every Saturday. As shown in Fig 1, CR was achieved with a step-down protocol [50], with daily food intake reduced to 90% of AL levels at 8 weeks of age, 75% of AL levels at 9 weeks of age and maintained at 60% of AL levels from 10 weeks onwards to 26 weeks of age. The 60% CR was carried on for 16 weeks in the CR groups. Mice housed in a laboratory generally prefer to eat more than enough for growth and life, so we concluded that intake at 60% CR permitted a healthy life. At 10 weeks of age, half of the mice in AL and CR groups were injected intravenously with 1 μg of AApoAII fibrils to induce amyloidosis (AL+F and CR+F groups) and the others were injected intravenously with an equal volume of vehicle (phosphate buffered saline, PBS) as control without induction of amyloidosis (AL+V and CR+V groups).

Every Monday evening (from approximately 18:00 to 19:30), all mice were weighed and their conditions examined for the following: wasting, injury, abscess, severe hair loss and hypo- or hyper-activities. The exclusion criteria included the following: (1) the presence of injuries, abscesses or other signs of illness; (2) in the AL groups, a slow increase in BW, i.e., <20 (AL+V) or <10 (AL+F) percentage increase at 26 weeks of age rather than the individual BW at 8 weeks of age; (3) in the CR groups, the presence of severe wasting, i.e., >20% decrease in BW compared to the individual BW at the beginning of 60% CR; and (4) unexpected death. According to the exclusion criteria, we selected 5 or 6 mice per group for analyses (S1 Table and S1 Fig).

### Ethics statements

All experiments using animals were performed with the approval of the Committee for Animal Experiments of Shinshu University and approved protocols were strictly adhered. Permit number: 250023 (in 2013).

### Induction of AApoAII amyloidosis

AApoAII amyloid fibrils were isolated from the liver of a 12-month-old R1.P1-*Apoa2c* mouse with heavy amyloid deposits as described previously [10,19,20]. In brief, AApoAII fibrils were isolated as a distilled water suspension using Pras’ method [51] and further purified by ultracentrifugation. The purified AApoAII fibrils (≥6 μg/μl) were stored in a –80°C freezer until used for induction of amyloidosis in mice. For induction of AApoAII amyloidosis, AApoAII fibrils were resuspended and diluted in ice cold PBS, and sonicated on ice according to our previous method [20]. We confirmed that there were abundant amyloid fibrils in the sonicated solution (S2 Fig). Mice were injected intravenously with a single dose of 1 μg of AApoAII fibrils in 100 μl PBS.

### Collection of plasma and tissue samples

On 10 February (first series) and 17 March (second series) in 2014 after a 16-week period of a 60% CR treatment, mice were sacrificed by cardiac puncture under deep diethyl ether anesthesia after overnight fasting (for 12–14 h), and plasma was collected. Several organs were collected, and half of them were kept in a -80°C freezer and the other half were fixed in 10% neutral buffered formalin to detect amyloid deposition.

### Detection of amyloid deposits

The formalin-fixed organs were embedded in paraffin and cut into 4-μm sections using standard procedures. Amyloid deposits were identified by polarizing light microscopy (LM) (Axioskop 2, Carl Zeiss Japan, Tokyo, Japan) to detect apple-green color birefringence in tissue sections stained with Congo red dye [16,17]. The intensity of amyloid deposits in each organ was determined semi-quantitatively using the amyloid score that was graded from 0 to 4 [10]. Each amyloid score was recorded as the average value graded by two observers who had no information regarding the examined tissue. The amyloid index (AI) was calculated as the average of the amyloid scores in seven organs (heart, liver, spleen, tongue, stomach, small intestine, and skin) representing the degree of amyloid deposit in each mouse [10,17,18].

AApoAII fibrils were also identified immunohistochemically using the horseradish peroxidase-labeled streptavidin-biotin method (DAKO, Glostrup, Denmark) with specific antisera against mouse ApoA-II and mouse AA, which were produced in our laboratory [10]. The antisera react specifically with ApoA-II and AA proteins, respectively on Western blot, and they also react specifically with AApoAII and AA amyloid fibrils, respectively, on both Western blot and immunohistochemical analyses [10,14,17,22,52]. Tissue sections were deparaffinized in Histoclear (National Diagnostics, GA, USA) and dehydrated in an alcohol series. Endogenous peroxidase activity was inhibited with 3% hydrogen peroxide/100% methanol, and the sections were blocked in 5% fetal bovine serum and 1% bovine serum albumin in PBS. The following primary antibodies were used: rabbit antisera against mouse ApoA-II (1:3000) and mouse AA (1:2000 dilution), both of which were diluted in blocking solution and incubated overnight at 4°C. The negative control was prepared by omission of the first antibody to validate the specificity of staining. For quantitative analysis, 3 areas in each liver and spleen section were randomly captured under ⅹ 200 magnification and the ratios of positively stained areas with anti-mouse ApoA-II antiserum to the whole section areas were calculated using an image processing program (NIH Image J software, version 1.61, MD, USA).

### Measurements of physiological and metabolic parameters

We determined the blood pressure and heart rate of each mouse at the beginning of the intervention of food intake (8 weeks of age) and at the end point of the intervention (26 weeks of age after 60% CR treatment for 16 weeks) using a computerized tail-cuff system (BP-98A-L, Softron Ltd., Tokyo, Japan). For analysis of blood glucose changes, overnight fasting glucose was determined from a sample obtained from the mouse tail vein; they were measured using a handheld glucose meter (Accu-Chek Aviva, Roche Diagnostic, Tokyo, Japan). On the same day of the measurement of fasting glucose levels, fasting mice were injected intraperitoneally with glucose (2 g/kg BW), and then blood from the tail vein was collected at arbitrary intervals for intraperitoneal glucose tolerance test (IGTT). Those data were expressed as the area under the curve over a 120 min period following the injection of glucose. Plasma total- and HDL-cholesterol levels and triglyceride levels after fasting were determined using quantitative assay kits based on an enzymatic procedure or by a modified heparin-manganese precipitation procedure [17,52].

### Measurements of ApoA-I and ApoA-II levels in plasma and in the liver of mice after intervention

We determined the concentrations of ApoA-I and ApoA-II proteins in plasma and the liver by Western blot and densitometry. Plasma samples (0.4 μl and 0.9 μl for ApoA-I and ApoA-II, respectively) were loaded at 15 mA for 6 h on a Tris-Tricine sodium dodecyl sulfate polyacrylamide gel (16.5% (w/v) acrylamide) for electrophoresis (SDS-PAGE). After electrophoresis, the gel was transferred to a PVDF membrane (Immobilon, 0.2 μm pore, Millipore Corp., MA, USA) using a semi-dry Western blot apparatus (WSE-4110, ATTO, Tokyo, Japan) at high voltage range for 20 min. After blocking in 3% skim milk (Morinaga Milk, Tokyo, Japan) for 1 h at room temperature (approximately 23 to 27°C), the membrane was then probed with polyclonal rabbit anti-mouse ApoA-I antiserum produced in our laboratory [53] (diluted 1:4000) and the polyclonal rabbit anti-mouse ApoA-II antiserum diluted 1:3000 in 3% skim milk in PBS containing 0.1% Tween-20 for 1 h at room temperature and overnight at 4°C. Then, the membranes were incubated for 1 h at room temperature with horseradish peroxidase-conjugated anti-rabbit IgG solution (Code #7074, Cell signaling Technology Inc., MA, USA) (1:3000) [14,52,53]. ApoA-I and ApoA-II were visualized with the enhanced chemiluminescence system using standard methods and were identified by molecular size markers (Kaleidoscope Polypeptide Standards 95790, Bio-Rad, CA, USA), which were loaded at the same time.

The liver samples were lysed in ice-cold RIPA lysis buffer (Santa Cruz Biotechnology, CA, USA) supplemented with Phos-STOP phosphatase inhibitor (Roche Science, Tokyo, Japan) and then homogenized. Fifty μg samples of protein extracts were loaded on Tris-Tricine/SDS-PAGE. ApoA-I, ApoA-II and β-Actin (BWT, MN, USA) (1:5000) were detected by Western blot and an enhanced chemiluminescence system as described above for plasma proteins. Protein contents of images were semi-quantitated using the NIH Image J software.

### Measurements of mRNA expression levels in the liver of mice after the intervention

Analysis of mRNA expression in the liver was performed using a method described previously [54]. Total RNA was extracted from the quick-frozen liver samples using TRIZOL Reagent (Invitrogen, CA, USA), after which the samples were treated with DNA-Free Reagent (Ambion, TX, USA) to remove contaminating DNA. Total RNA was reverse-transcribed at 10 ng/μl final concentration using the High Capacity cDNA Reverse Transcription Kit with random primers (Applied Biosystems, CA, USA). The cycling parameters for reverse transcriptase-polymerase chain reaction (RT-PCR) amplification were as follows: initial denaturation for 1 min at 94°C, followed by 23–30 cycles of 30 sec at 94°C, 30 sec at 60°C, and 45 sec at 72°C. Quantitative real-time RT-PCR analysis was carried out using a sequence detection system (ABI PRISM 7500, Applied Biosystems, CA, USA) with SYBR Green (TaKaRa Bio, Tokyo, Japan). Gene expression was normalized to the gene for β-actin as a housekeeping gene. The forward and reverse primer sequences are listed in S2 Table.

### Chemical reagents

Unless otherwise specified, chemical reagents in the experiments were obtained from Wako Pure Chemical Industries, Ltd. (Osaka, Japan).

### Statistical analysis

We used the R software package (The R Development Core Team, Vienna University of Economics and Business, Vienna, Austria). P values <0.05 were regarded as statistically significant.

Before starting the intervention, i.e., the baseline body condition, we examined any significant differences in BWs, blood pressures, heart rates, fasting blood glucose levels and areas under the curve calculated from the data of IGTT among the four groups (AL+V, CR+V, AL+F, and CR+F) with the Tukey-Kramer method for multiple comparisons. Using this method, we also examined any significant differences in changes of weekly BWs, in physical and blood biochemical parameters, contents of ApoA-I and ApoA-II proteins by Western blot, and mRNA levels by real-time RT-PCR after the intervention among the four groups. We used the paired Student’s *t*-test for any significant differences in physical and glucose metabolic parameters between the baseline and values at the end of the intervention.

The Mann-Whitney *U*-test was used to analyze the averages of amyloid score and AI for amyloid deposits between the AL+F and CR+F groups. Data from the immunohistochemical images in the liver and spleen were analyzed for the suppressive abilities of the CR treatment for amyloid deposits by the non-paired Student’s *t*-test.

## Results

### CR treatment suppressed BW increases within the growth period in young mice

To avoid unexpected death by sudden and severe CR, we fed mice with a step-down protocol (Fig 1). This protocol was performed as follows: 90% CR the first week, 75% CR the second week, and 60% CR the third week, a level that was continued for 16 weeks.

We performed the interventions with 32 female mice in two separate series (N = 4 per series), and then we selected and analyzed 21 mice among them (N = 6 (CR+F) or 5 (others)) according to the exclusion criteria (S1 Table and S1 Fig). The incidence of excluded events was not different among the four groups. We confirmed that there were no significant differences in the BWs of the examined mice at the beginning of the interventions among the four groups (BWs (g, means ± S.D.): 22.10 ± 0.80 (AL+V), 21.66 ± 1.02 (CR+V), 22.12 ± 1.66 (AL+F), and 22.66 ± 0.58 (CR+F)). After the intervention, the mean (± S.D.) BWs were 26.93 ± 1.52 (AL+V), 21.35 ± 1.14 (CR+V), 26.11 ± 1.73 (AL+F) and 19.80 ± 1.21 (CR+F) grams. BWs in the CR groups (CR+V and CR+F) were unchanged or decreased during the CR treatment although it increased in the AL groups (AL+V and AL+F) during the same period (Fig 1). After 2 weeks of the 60% CR treatment, a significant difference was observed in the change of BWs between AL and CR groups, and continued during the CR treatment. The significant differences in the change of BWs were independent of the induction of amyloidosis.

### CR treatment prevented systemic AApoAII amyloid deposits in mice

**Fig 2:**
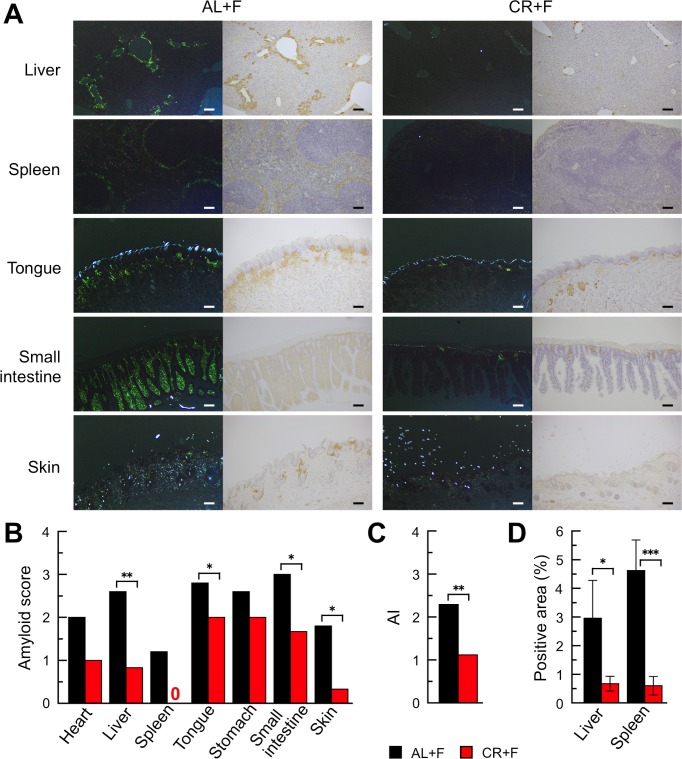
CR treatment suppressed systemic progression of AApoAII deposits in mice. A single administration of 1 μg AApoAII fibrils was given to female mice. After 16 weeks, amyloid deposits were detected. (A) LM images of AApoAII deposits in several organs in mice. *Left-hand panels in each group*, Amyloid deposits were identified by green birefringence in Congo red-stained sections using polarized LM. *Right-hand panels in each group*, AApoAII deposits were confirmed immunohistochemically with anti-ApoA-II antiserum. Each scale bar indicates 100 μm. (B and C) The amyloid scores in several organs and the AI in whole bodies in mice. Each column represents the means (N = 5 (AL+F) and 6 (CR+F)). *p<0.05, **p<0.01 (non-parametric Mann-Whitney *U* test). (D) Comparison with positive areas of amyloid deposits in the liver and spleen using the immunohistochemical method with anti-ApoA-II anti-serum. Three areas in each liver and spleen section were randomly captured under x 200 magnification and the positive areas were calculated with the Image J system. Each column and bar represents the mean ± S.D. (N = 5 (AL+F) and 6 (CR+F)). *p<0.05, **p<0.001 (non-paired Student’s *t* test)**.**

As shown in Fig 1, we injected mice with a single dose of 1 μg AApoAII fibrils at the beginning of the 60% CR treatment. After 16 weeks, we collected several organs for the comparison of amyloid deposits between AL and CR groups. First, we evaluated amyloid deposits by the presence of apple-green color birefringence in Congo red-stained tissue under polarized LM. Although no amyloid deposits were observed in any organs in the control groups injected with PBS (AL+V and CR+V), areas of amyloid deposition were detected systemically in the amyloidosis-induced mice (AL+F and CR+F) (Fig 2A and S3 Table). Under polarized LM, the apple-green colored areas in Congo red-stained sections from the CR+F mice were obviously smaller than those from the AL+F group. Those positive areas were stained with anti-ApoA-II antiserum under normal LM. AApoAII amyloid deposits were confirmed by immunohistochemistry, and no AA was deposited in the same areas (S3 Fig). According to semi-quantitative analyses using the sections stained with Congo red dye, the grades of amyloid deposits were significantly lower in the liver, tongue, intestine and skin of the CR+F group compared with the AL+F group (Fig 2B). The intensities of the amyloid deposits in the whole body (amyloid index, AI) were significantly lower in the CR+F group than in the AL+F group **(**Fig 2C). Next, we sought to confirm the suppressive effects of CR treatment on AApoAII amyloid deposition. Towards that end, we used an image processing program to assess the areas of amyloid deposition in microscopic images of the liver and spleen stained immunohistochemically with anti-ApoA-II antiserum (Fig 2A). The areas containing AApoAII deposits in both organs in the CR+F group were significantly smaller than those in the AL+F group (Fig 2D**)**.

### CR treatment changed blood glucose levels but not plasma lipid parameters in mice

**Fig 3:**
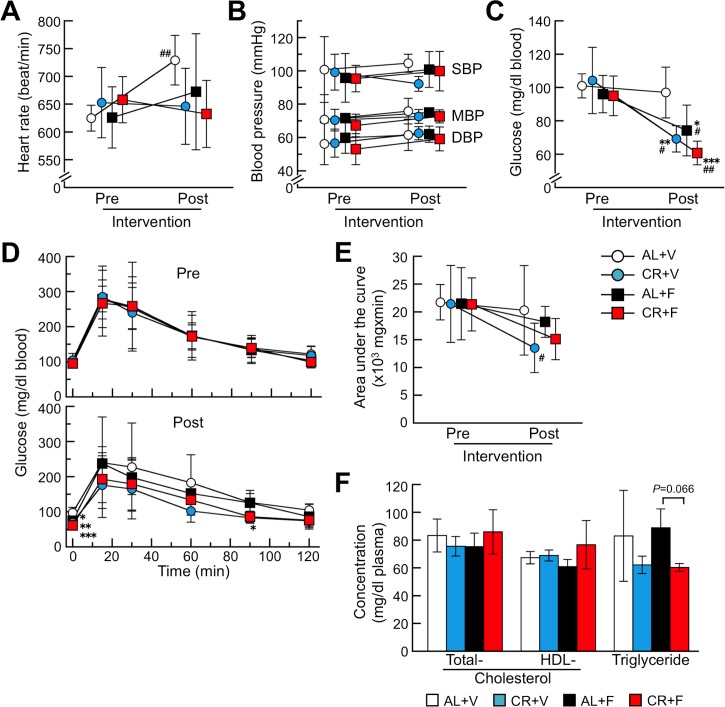
CR treatment might improve glucose metabolism. These panels show the heart rates (A), blood pressures (B), fasting blood glucose levels (C) and changes of blood glucose and areas under the curve determined by the IGTT (D and E, respectively) before (pre) and after (post) the intervention. Values of plasma lipids after the intervention were shown in (F). Each symbol or column and bar represents the mean ± S.D. (N = 6 (CR+F) and 5 (others)). None of the baseline (pre) parameters were significantly different among the four groups (Tukey-Kramer method). *p<0.05, **p<0.01, ***p<0.001 vs. the AL+V group (Tukey-Kramer method for multiple comparison at corresponding time). #p<0.05, ##p<0.01 vs. the each baseline (paired Student’s *t* test).

Physiological activities of the heart (heart rate and blood pressure) did not change after the intervention (Fig 3A and 3B). Heart rates in the AL+V group after the intervention increased over their baseline, but they were not significantly different than those in the other groups. Fasting blood glucose levels were significantly decreased after the intervention compared with their baseline, but not in the AL+V group (Fig 3C). According to IGTT and the data calculated from the areas under the curve, glucose tolerance tended to be improved after CR treatment (Fig 3D and 3E). Plasma total- and HDL-cholesterol levels after the intervention were not significantly different among the four groups, but triglyceride levels tended to be lower in the CR treatment groups (Fig 3F).

### CR treatment decreased the levels of ApoA-II protein in mouse plasma and the liver

**Fig 4:**
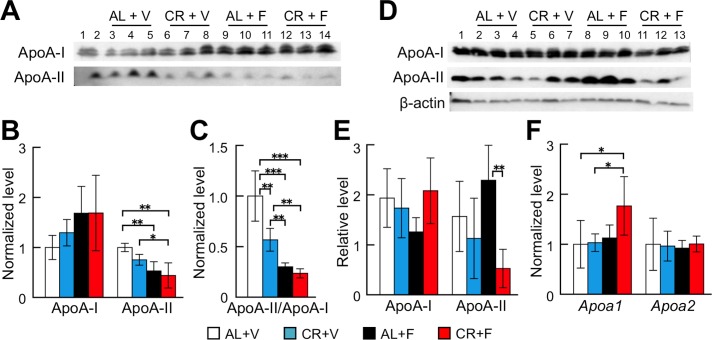
CR treatment reduced the ApoA-II level in plasma. Concentrations of ApoA-I and ApoA-II proteins in plasma (A-C) and in the liver (D and E) after the CR intervention were determined by Western blot and densitometry, and mRNA expression levels of ApoA-I and ApoA-II in the liver were determined by quantitative real-time PCR (F). (A) Lane 1 or 2, the pooled plasmas of female R1.P1-*Apoa2c* mice at 2 months of age (N = 4) that did not have AApoAII amyloid deposits, as the positive control of these proteins. Lanes 3–5, 6–8, 9–11 and 12–14, plasmas (N = 3) selected at random from the AL+V, CR+V, AL+F, and CR+F groups, respectively. (D) Lane 1, pooled plasma. Lanes 2–4, 5–7, 8–10 and 11–13, samples from the livers of the same mice used in (A). (B-D, F) Each column and bar represent the mean ± S.D. (N = 5 (AL+F) and 6 (CR+F)). *p<0.05, **p<0.01, ***p<0.001 (Tukey-Kramer method for multiple comparison).

In order to quantitate the changes of ApoA-I and ApoA-II levels after the CR treatment and/or the induction of amyloidosis, we determined those levels in plasma and the liver after intervention (Fig 4). Plasma ApoA-I levels tended to increase in the amyloidosis-induced groups (AL+F and CR+F), but no significant difference was observed between the groups (Fig 4A and 4B). Plasma ApoA-II levels decreased significantly in mice regardless of the CR treatment (AL+F and CR+F) compared with those in the AL+V group. To further confirm the relationship between ApoA-I and ApoA-II, the ratios of ApoA-II to ApoA-I were calculated (Fig 4C). The ApoA-II/ApoA-I ratio was significantly decreased by CR (CR+V) and further decreased by the induction of amyloidosis (AL+F and CR+F). In the liver, ApoA-I contents were not significantly different among the groups (Fig 4D and 4E). ApoA-II contents tended to be higher in the AL+F group, which had the greatest deposits of AApoAII fibrils in the liver among all of the groups. Interestingly, the ApoA-II contents in the livers of the CR+F group were lower than those in the AL+F group even though there were mild depositions of AApoAII fibrils in the liver of the CR+F group. The expression levels of *Apoa1* and *Apoa2* mRNA did not change after CR treatment and amyloidosis induction (Fig 4F).

### CR treatment might improve chronic inflammation, stress response, and mitochondrial function

**Fig 5:**
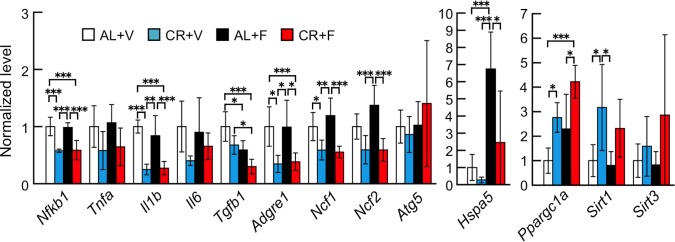
CR treatment might reduce chronic inflammation, stress response, and unfolded protein response induced by amyloid deposits and upregulate mitochondrial function. Using quantitative real-time RT-PCR, we determined mRNA expression levels of the following genes in the liver after the intervention: inflammation-related genes (*Nfkb1*, *Tnfa*, *Il1b*, *Il6*, and *Tgfb1*), macrophage marker gene (*Adgre1*), stress response-related genes (*Ncf1* and *Ncf2*), autophagy-related gene (*Atg5*), unfolded protein response sensor gene (*Hspa5*) and mitochondrial function-related genes (*Ppargc1a*, *Sirt1*, and *Sirt3*). Each column and bar represents the mean ± S.D. (N = 6 (CR+F) and 5 (others)). *p<0.05, **p<0.01, ***p<0.001 (Tukey-Kramer method for multiple comparison).

To further investigate the mechanisms underlying the preventive effects of CR on the progress of amyloidosis, we determined the mRNA levels of genes involved in chronic inflammation such as NF-κB (*Nfkb1*), TNF-α (*Tnfa*), IL-1β (*Il1b*), IL-6 (*Il6*), TGF-1β (*Tgfb1*) and F4/80 (*Adgre1*), a macrophage marker in the liver (Fig 5). The mRNA levels of *Nfkb1*, *Il1b* and *Adgre1* were significantly decreased in the CR groups (CR+V and CR+F). The levels of *Tnfa* and *Il6* tended to be less than those of the AL groups, but all levels of the CR groups were lower regardless of the amyloidosis-induction. *Tgfb1* levels were lower in the amyloidosis-induction groups, but not different between the AL cohort and those receiving CR treatment. We also determined expression levels of oxidative stress-related factors, such as P47phox (*Ncf1*) and P67phox (*Ncf2*). They were decreased in the CR groups but not changed in animal showing amyloidosis. For other stress responses, we determined mRNA expression levels of ATG5 (*Atg5*, an autophagy-related factor) and BIP (*Hspa5*, a key molecule for the unfolded protein response). The expression levels of *Atg5* were not significantly different among any of the groups, and the levels of *Hspa5* were upregulated in the amyloidosis-induced group without CR treatment (AL+F) but repressed by CR treatment (CR+F). In contrast, the CR treatment significantly upregulated the expression levels of PGC-1α (*Ppargc1a*), a major factor that controls mitochondrial biogenesis and maintenances of mitochondria, and SIRT1 (*Sirt1*), a well-known longevity gene and a regulator of PGC-1α. Treatment also tended to upregulate the expression levels of SIRT3 (*Sirt3*), an anti-oxidative stress factor in mitochondria.

## Discussion

In our previous report, we demonstrated that an extended 60% CR treatment starting at 4 weeks of age increased the lifespan and prevented spontaneous deposition of AApoAII fibrils in the liver, skin, spleen and testis of senile AApoAII amyloidosis-susceptible mice [48]. In that paper, we hypothesized that the slowdown of the aging progress during the CR treatment might reduce the incidence and progression of AApoAII amyloidosis, but we did not know the biological mechanisms by which CR decelerated aging and prevented amyloidosis. In this study, we performed short-term 60% CR treatment for 16 weeks in mice in which we had experimentally induced AApoAII amyloidosis. We focused on the benefits of CR treatment and analyzed the biochemical and molecular mechanisms by which CR treatment reduced amyloidosis.

Subjecting mice to a 60% CR for 16 weeks was not a severe regimen, as life was maintained in the CR groups. Actually, CR-treated mice lived similar to the AL groups. However, the BWs in the CR groups were significantly lower than those in the AL groups (Fig 1, S1 Table and S1 Fig). There were no significant differences in the BWs between the CR+V and CR+F groups. We previously observed no loss of BW in female SAMR1 mice treated with a 60% CR [48]. The SAMR1 strain possesses the genetic background of R1.P1-*Apoa2c* congenic mice that we examined in the present experiment. Thus, we propose that the present CR strategy suppressed the BW increase in mice during the period of growth. Moreover, the physiological data including circulatory functions and lipid contents in plasma were not different among the four groups (Fig 3).

We found that the CR treatment significantly suppressed systemic induction and/or progression of AApoAII amyloidosis (Fig 2). We suggest that suppressing the levels of amyloid precursor proteins in the body might be a good first step in preventing amyloid deposition in almost all amyloidoses [3,55]. Conversely, an increase in plasma concentrations of amyloid precursor protein accelerates amyloidosis. For example, in the case of *Apoa2c* transgenic mice that are AApoAII amyloidosis-susceptible, the plasma ApoA-II levels in homozygous transgenic mice were 1.57- or 1.26-fold greater than in wild-type or heterozygous littermates, respectively [14]. Furthermore, AApoAII fibril deposition was accelerated in almost every organ depending on the plasma levels of ApoA-II protein [14]. We found that the plasma ApoA-II concentrations in the CR+V group tended to be lower than those in the AL+V group without CR treatment. Moreover, the ApoA-II concentrations in the groups with amyloid deposits (AL+F and CR+F) were also significantly lower than those in the AL+V group (Fig 4B). Plasma ApoA-II concentrations might be decreased by the transfer of ApoA-II proteins into amyloid deposits throughout the body. It is also possible that the accelerated turnover rate of plasma ApoA-II might reduce the concentration of ApoA-II [56]. In fact, a decrease in the concentration of SAA after amyloid deposition was observed in experimental AA amyloidosis [57]. In the liver, the primary site of ApoA-II synthesis, the protein contents did not differ between AL+V and CR+V groups (Fig 4D and 4E). It was also reported that the glucose-induced transcription of the *Apoa2* gene and the human plasma concentration of ApoA-II correlated with the blood glucose level [58]. The fasting glucose levels after CR treatment were decreased significantly compared with their baseline values (Fig 3C), but there were no differences in the expression levels of *Apoa2* mRNA for all examined groups (Fig 4F).

Although various epigenetic factors regulate the progress and toxicity of amyloidosis, nutritional control might be one of the most promising treatments to prevent age-related amyloidosis in countries where the excessive intake of calories causes pro-pathologic conditions such as metabolic syndrome. In AApoAII amyloidosis, in which the precursor protein ApoA-II is a major apolipoprotein of HDL, dietary fat modulates ApoA-II metabolism and amyloid deposition. For example, olive oil- and safflower oil-containing diets suppressed accelerated amyloid deposition whereas fish oil exacerbated it [59,60]. Moreover, ApoA-I-deficient mice showed accelerated AApoAII amyloid deposition by redistribution of ApoA-II in HDL and age-related increases in ApoA-II levels [52]. Since many amyloid-related proteins including SAA, ApoA-I, ApoA-IV, ApoC-II, ApoE, clusterin, and Aβ are associated with HDL [61], nutritional treatments modifying the metabolism of HDL might prevent several types of amyloidosis. In this study, plasma lipid parameters were not different among the four groups (Fig 3F). From our results and previous reports, we propose that the main cause of the suppression of amyloid deposition in the CR group can be attributed to the decreased level of plasma amyloidogenic ApoA-II protein. Reduced secretion, accelerated turnover rate or other mechanisms leading to the reduction of ApoA-II levels should be elucidated in future studies.

Many studies have shown that CR can reduce chronic inflammation, increase mitochondrial metabolism and biogenesis, inhibit oxidative stress and promote autophagy and other processes [27,28,30,62]. It has been postulated that the sirtuin families, NAD-dependent deacetylases and nutrient sensors might be master factors that control the beneficial effects of CR [63–65]. SIRT1 protein was reported to increase expression levels and enhance the function of ATG5 [46,66]. ATG5 is an essential modulator of autophagy initiated by CR, and it is associated with the extension of lifespan [30,31]. However, in the present study, the mRNA expression levels of *Atg5* were not different among the four groups (Fig 5). SIRT1 protein was also reported to increase expression levels and strengthen the function of PGC-1α [54,67]. PGC-1α protein is important in the regulation of mitochondrial biogenesis and respiration. Furthermore, activated mitochondrial function might reduce oxidative stress, and SIRT1 activates PGC-1α in the liver in response to fasting signals [67]. SIRT1 protein is essential for extension of lifespan, and in the absence of SIRT1 protein, CR cannot extend the lifespan of mice [65]. It was also reported that the activation of neuronal SIRT1 was a possible mechanism underlying the protective effects of CR against the pathology of Alzheimer’s disease [44,68]. We previously reported that activation of SIRT1 (by daily supplementation of the reduced form of coenzyme Q10 (ubiquinol-10)) increased mitochondrial biogenesis and respiration through an increase in the active form and activation of PGC-1α in the liver of SAMP1 mice [54]. We found here that CR treatment significantly decreased fasting levels of glucose and tended to improve glucose metabolism compared with baseline values (Fig 3C–3E). Furthermore, CR significantly increased the gene expression levels of *Sirt1* and *Ppargc1a* and tended to increase the gene expression levels of *Sirt3*, which controls the activities of several molecules for respiration and anti-oxidation in mitochondria. Moreover, it was reported that the anti-inflammatory action of SIRT1 occurs through inhibition of NF-κB and TNF-α [64]. Here, CR repressed the expression of genes related to chronic inflammation (Fig 5). From these findings, we suggest that the improvement of glucose metabolism during CR treatment is at least partly due to the increased expression levels of *Sirt1* and *Ppargc1a*.

In mice bearing skin cancer, inflammation accompanied by cellular proliferation was reduced when CR treatment upregulated ApoA-I protein [69]. In contrast, we found that CR intervention did not alter either the gene expression levels or the protein content of ApoA-I in the livers of mice without AApoAII amyloidosis (Fig 4E and 4F). From these findings, we propose that CR treatment might improve the inflammatory state and mitochondrial functions via the SIRT1-PGC-1α pathway in mice with amyloidosis. Furthermore, the relationship between inflammation and proteoglycans in extracellular matrices, e.g., heparin sulfate, might regulate amyloid fibril deposition [70,71]. Macrophages play important roles in the progression and toxicity of amyloid deposition [72–74]. In particular, a recent report found that macrophages in a transthyretin amyloidosis mouse model were activated by daily supplementation of curcumin, which is known to be an anti-oxidant, an anti-inflammatory agent and also a chelator of amyloid-associated metal ions, thereby reducing amyloid fibril aggregation and/or neurotoxicity and internalized and degraded amyloid aggregates [74]. Interestingly, we found that the expression levels of the macrophage marker F4/80 (*Adgre1*) were significantly suppressed in CR-treated groups (Fig 5). We believe that the reduction of AApoAII amyloid deposits in the CR-treated mice is partly due to the decreased circulation of amyloidogenic ApoA-II protein rather than the phagocytosis of amyloid aggregates by activated macrophages.

We also found that the P47phox (*Ncf1*) and P67phox (*Ncf2*) genes were suppressed (Fig 5). They are oxidative stress markers, and oxidative stress is observed during aging and in numerous age-related diseases including amyloidosis [75,76]. CR treatment decreased oxidative damage in the majority of the reports, although the precise mechanisms have not been well elucidated [62]. Furthermore, the increased expression of BIP (*Hspa5*) associated with amyloid deposits in the AL+F group was reduced in the CR+F group and was associated with suppression of amyloid deposits. BIP is a key molecule in the unfolded protein response and is upregulated in the liver with AApoAII amyloid deposits [77]. From our data, CR treatment might lessen amyloid deposition by reducing oxidative stress and improving the unfolded protein response. These results suggested that the beneficial effects of CR are indeed complex. It is currently difficult to pinpoint the direct effects of CR that suppress amyloid deposition. This is due to the fact that there might be a limitation on the control of food intake when mice are housed in groups. Further investigations using CR treatment with more strict controls on the caloric intake of individual animals will be performed to better elucidate the precise mechanisms that suppress the progression of amyloidosis.

In conclusion, CR treatment might induce systemic responses that slow the progress of mouse AApoAII amyloidosis by modifying the metabolism of precursor ApoA-II protein. However, additional beneficial effects of CR treatment may have preventive effects on amyloidosis. These findings should provide useful guidance for determining the cause of amyloidosis and developing effective preventive treatments.

## Supporting information

S1 Table Raw data describing prepared and examined mice. (DOCX)

S2 Table The specific primers for real-time RT-PCR. (DOCX)

S3 Table Raw data for amyloid deposition in all mice with or without the injection of AApoAII amyloid fibrils. (DOCX)

S1 Fig Individual body weights and selected or excluded mice: raw data. We performed two experiments (first and second series) (N = 4 for preparation in each series). Mice were weighed every Monday evening and judged according to the exclusion criteria described in Materials and Methods. Then, mice were selected for analyses (N = 5 or 6 per group). (See S1 Table).
(TIF)

S2 Fig TEM image of the solution used for induction of AApoAII amyloidosis. Frozen AApoAII amyloid fibrils isolated from the liver of a mouse with severe AApoAII amyloidosis were thawed on ice, resuspended and diluted in ice cold PBS. The solution was sonicated on ice and was immediately injected into the tail vein of each mouse. TEM image shows the AApoAII solution after sonication. There were abundant amyloid fibrils with characteristic structures in the solution. Scale bar indicates 100 nm.
(TIF)

S3 Fig Lack of AA fibril deposition in induced AApoAII amyloidosis. Female mice were given a single administration of 1 μg AApoAII fibrils. After 16 weeks, amyloid deposits were assessed by the protocol described in Materials and Methods. *Left-hand panels*, Congo red stained sections of the spleen, tongue, and small intestine using polarized LM. They were typical images from the corresponding slices with amyloid deposits shown in Fig 2A. *Middle and Right-hand panels*, Immunohistochemical stained slices with anti-ApoA-II or anti-AA antisera, respectively. Each scale bar indicates 100 μm in width.
(TIF)

## Abbreviations

AA: amyloid A
AApoAII: amyloid fibrils derived from ApoA-II
AI: amyloid index
AL: *ad* *libitum*
Apo: apolipoprotein
BW: body weight
CR: caloric restriction
IGTT: intraperitoneal glucose tolerance test
HDL: high-density lipoprotein
LM: light microscopy
PAGE: polyacrylamide gel electrophoresis
PBS: phosphate buffered saline
PGC-1α: peroxisome proliferator-activated receptor γ coactivator 1α
RT-PCR: reverse transcriptase-polymerase chain reaction
SAMP1: Senescence-Accelerated mouse Prone 1
SDS: sodium dodecyl sulfate
SIRT1: sirtuin 1
