# Supplementary material for: Caloric restriction reduces the systemic progression of mouse AApoAII amyloidosis
Source: PLoS One. 2017 Feb 22;12(2):e0172402. doi: 10.1371/journal.pone.0172402 (PMC5321440; doi:10.1371/journal.pone.0172402)
Supplement: S1 Table — (DOCX) [file pone.0172402.s001.docx]

**S1 Table. Raw data describing prepared and examined mice.**

| Group | Prepared mice | | Examined mice | Excluded mice | | | | | |
| --- | --- | --- | --- | --- | --- | --- | --- | --- | --- |
|  | Number | BW^1^ (g) | Number | Death | | Dropout | | Others | |
|  |  |  |  | Number | Reason | Number | Reason | Number | Reason |
| First series (Birthday; 10–12 August in 2013) | | | | | |  |  |  |  |
| AL+V | 4 | 22.20 ± 0.92 | 3 | 0 | - | 0 | - | 1 | Slow increase on BW^4^ |
| CR+V | 4 | 23.13 ± 2.77 | 1 | 1 | Unknown^2^ | 2 | Severe decrease on BW^3^ | 0 | - |
| AL+F | 4 | 22.39 ± 1.75 | 2 | 0 | - | 0 | - | 2 | Slow increase on BW |
| CR+F | 4 | 22.39 ± 0.67 | 3 | 1 | Unknown | 0 | - | 0 | - |
|  |  |  |  |  |  |  |  |  |  |
| Second series (Birthday; 13–18 September in 2013) | | | | | |  |  |  |  |
| AL+V | 4 | 22.14 ± 0.46 | 2 | 1 | Unknown | 0 | - | 1 | Slow increase on BW^3^ |
| CR+V | 4 | 22.09 ± 0.38 | 4 | 0 | - | 0 | - | 0 | - |
| AL+F | 4 | 22.98 ± 1.41 | 3 | 0 | - | 0 | - | 1 | Slow increase on BW |
| CR+F | 4 | 22.62 ± 0.55 | 3 | 0 | - | 1 | Severe decrease on BW | 0 | - |

1. Body weight (BW) at 8 weeks of age. Data represents the mean ± S.D. (N = 4). There were no significant differences in the BWs among the four groups in each series (Tukey-Kramer method for multiple comparisons).

2. The cause of death was not determined, although the dead mice were subjected to necropsices.

3. The grade of the ‘severe’ decrease of BW decreased more than 20% of BW at the beginning of 60% CR.

4. The grade of the ‘slow’ increase of BW at 26 weeks of age increased less than 20 (AL+V) or 10 (AL+F) percent of BW at 8 weeks of age.
