# Supplementary material for: Caloric restriction reduces the systemic progression of mouse AApoAII amyloidosis
Source: PLoS One. 2017 Feb 22;12(2):e0172402. doi: 10.1371/journal.pone.0172402 (PMC5321440; doi:10.1371/journal.pone.0172402)
Supplement: S2 Table — (DOCX) [file pone.0172402.s002.docx]

**S2 Table. The specific primers for real-time RT-PCR**

| Gene Primer sequences (5’–3’) |
| --- |
| *Apoa1* Forward:  *GTGGCTCTGGTCTTCCTGAC* |
| Reverse: *ACGGTTGAACCCAGAGTGTC* |
| *Apoa2* Forward:  *GCCTGTTCACTCAGTACTTTCAG* |
| Reverse: *CAGACTAGTTCCTGCTGACC* |
| *Nfkb1* Forward:  *ATGGCAGACGATGATCCCTAC* |
| Reverse: *TGTTGACAGTGGTATTTCTGGTG* |
| *Τnf* Forward:  *ACGGCATGGATCTCAAAGAC* |
| Reverse: *AGATAGCAAATCGGCTGACG* |
| *Il1b* Forward:  *TCTCACAGCAGCACATCAAC* |
| Reverse: *TCGTTGCTTGGTTCTCCTTG* |
| *Il6* Forward:  *CCCAATTTCCAATGCTCTCC* |
| Reverse: *TGAATTGGATGGTCTTGGTCC* |
| *Tgfb1* Forward:  *CCCGAAGCGGACTACTATGC* |
| Reverse: *TAGATGGCGTTGTTGCGGT* |
| *Adgre1* Forward:  *GATGAATTCCCGTGTTGTTGGT* |
| Reverse: *ACATCAGTGTTCCAGGAGACACA* |
| *Ncf1* Forward:  *ATCCTATCTGGAGCCCCTTGA* |
| Reverse: *CACCTGCGTAGTTGGGATCC* |
| *Ncf2* Forward:  *CAGACCCAAAACCCCAGAAA* |
| Reverse: *AAAGCCAAACAATACGCGGT* |
| *Atg5* Forward:  *AGAGTCAGCTATTTGACGTTGG* |
| Reverse: *TGGACAGTGTAGAAGGTCCTTTT* |
| *Hspa5*  Forward:  *ACCCCGAGAACACGGTCTT* |
| Reverse: *TGCCCACCTCCAATATCAACT* |
| *Ppargc1a* Forward:  *TCACCACCGAAATCCTTA* |
| Reverse: *GGTGTCTGTAGTGGCTTGAT* |
| *Sirt1*  Forward:  *CCTTGGAGACTGCGATGTTA* |
| Reverse: *GTGTTGGTGGCAACTCTGA* |
| *Sirt3* Forward:  *TACAGGCCCAATGTCACTCA* |
| Reverse: *ACAGACCGTGCATGTAGCTG* |
| *Actb* Forward:  *GACAGGATGCAGAAGGAGATTACT* |
| Reverse: *TGATCCACATCTGCTGGAAGGT* |
