# Supplementary material for: Caloric restriction reduces the systemic progression of mouse AApoAII amyloidosis
Source: PLoS One. 2017 Feb 22;12(2):e0172402. doi: 10.1371/journal.pone.0172402 (PMC5321440; doi:10.1371/journal.pone.0172402)
Supplement: S3 Table — (DOCX) [file pone.0172402.s003.docx]

**S3 Table. Raw data for amyloid deposition in all mice with or without the injection of AApoAII amyloid fibrils.**

| Group | Number | CR | AApoAII  -injection | Amyloid score (Average) | | | | | | | | | AI |
| --- | --- | --- | --- | --- | --- | --- | --- | --- | --- | --- | --- | --- | --- |
|  |  |  |  | Heart | Liver | Spleen | Tongue | Stomach | Small | Skin | Lung | Kidney |  |
|  |  |  |  |  |  |  |  |  | intestine |  |  |  |  |
| AL+V | 5 | − | − | 0 | 0 | 0 | 0 | 0 | 0 | 0 | 0 | 0 | 0 |
| CR+V | 5 | + | − | 0 | 0 | 0 | 0 | 0 | 0 | 0 | 0 | 0 | 0 |
| AL+F | 5 | − | + | 2.00 | 2.60 | 1.20 | 2.80 | 2.60 | 3.00 | 1.80 | 2.00 | 0 | 2.29 |
| CR+F | 6 | + | + | 1.00 | 0.83 | 0 | 2.00 | 2.00 | 1.67 | 0.33 | 1.50 | 0 | 1.12 |

AI; the average of the amyloid score values in seven organs (heart, liver, spleen, tongue, stomach, small intestine, and skin) representing the degree of amyloid deposit in each mouse.
