# Supplementary figures and images for: Caloric restriction reduces the systemic progression of mouse AApoAII amyloidosis
Source: PLoS One. 2017 Feb 22;12(2):e0172402. doi: 10.1371/journal.pone.0172402 (PMC5321440; doi:10.1371/journal.pone.0172402)

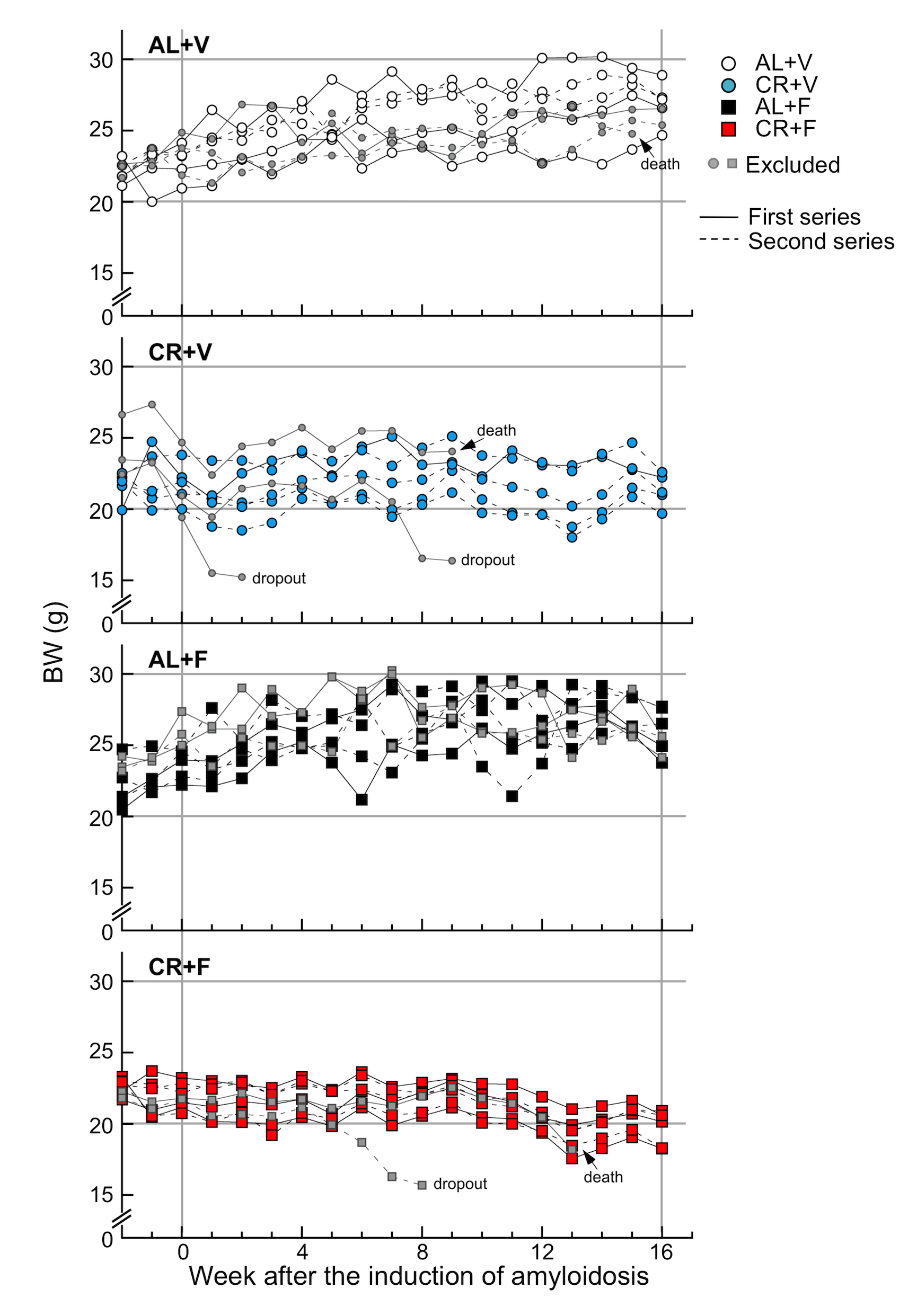

Supplement: S1 Fig — We performed two experiments (first and second series) (N = 4 for preparation in each series). Mice were weighed every Monday evening and judged according to the exclusion criteria described in Materials and Methods. Then, mice were selected for analyses (N = 5 or 6 per group). (See S1 Table). (TIF) [file pone.0172402.s004.tif]

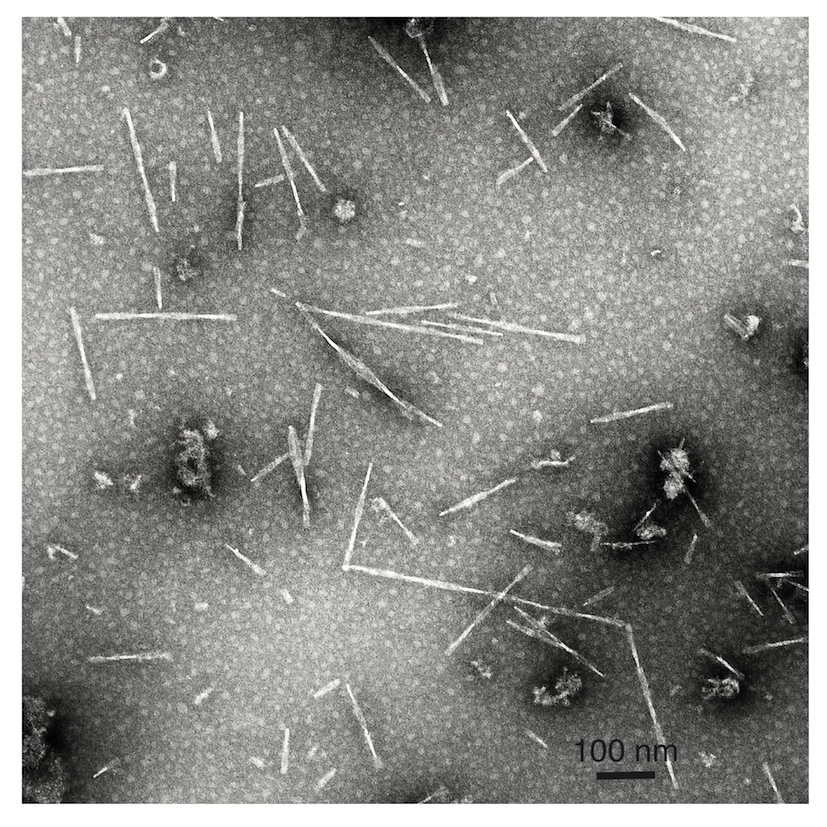

Supplement: S2 Fig — Frozen AApoAII amyloid fibrils isolated from the liver of a mouse with severe AApoAII amyloidosis were thawed on ice, resuspended and diluted in ice cold PBS. The solution was sonicated on ice and was immediately injected into the tail vein of each mouse. TEM image shows the AApoAII solution after sonication. There were abundant amyloid fibrils with characteristic structures in the solution. Scale bar indicates 100 nm. (TIF) [file pone.0172402.s005.tif]

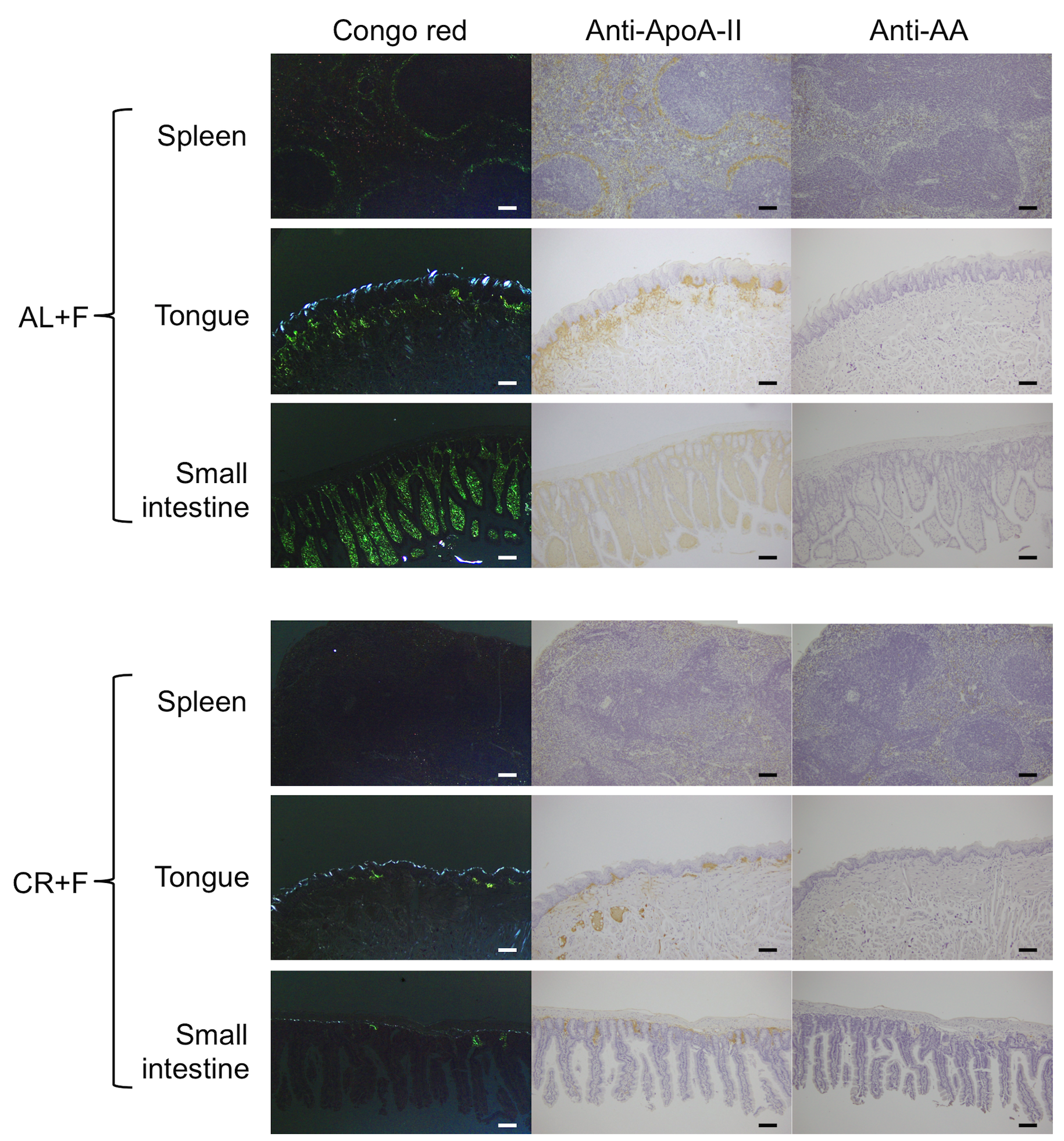

Supplement: S3 Fig — Female mice were given a single administration of 1 μg AApoAII fibrils. After 16 weeks, amyloid deposits were assessed by the protocol described in Materials and Methods. Left-hand panels, Congo red stained sections of the spleen, tongue, and small intestine using polarized LM. They were typical images from the corresponding slices with amyloid deposits shown in Fig 2A. Middle and Right-hand panels, Immunohistochemical stained slices with anti-ApoA-II or anti-AA antisera, respectively. Each scale bar indicates 100 μm in width. (TIF) [file pone.0172402.s006.tif]
